# Holistic physical exercise training improves physical literacy among physically inactive adults: a pilot intervention study

**DOI:** 10.1186/s12889-019-6719-z

**Published:** 2019-04-11

**Authors:** Peter Holler, Johannes Jaunig, Frank-Michael Amort, Silvia Tuttner, Kathrin Hofer-Fischanger, Dietmar Wallner, Helmut Simi, Alexander Müller, Mireille Nicoline Maria van Poppel, Othmar Moser

**Affiliations:** 1 Institute of Health Management in Tourism, FH JOANNEUM, University of Applied Sciences, Bad Gleichenberg, Austria; 2 Department of Public Health, Institute for Health Promotion and Disease Prevention (IfGP), Graz, Austria; 3 Institute of Sports Science, University of Graz, Graz, Austria; 4 Cardiovascular Diabetology Research Group Division of Endocrinology and Diabetology Department of Internal Medicine Medical University of Graz, Graz, Austria

**Keywords:** Physical literacy, Physical exercise training, Physical inactivity, holistic, Physical exercise intervention

## Abstract

**Background:**

Physical literacy (PL), given as a multidimensional construct, is considered a person’s capacity and commitment to a physically active lifestyle. We investigated the effect of a holistic physical exercise training on PL among physically inactive adults.

**Methods:**

A non-randomised controlled study was conducted. Thirty-one physically inactive adults in the intervention group (IG; 81% females, 44 ± 16 years) participated in a holistic physical exercise training intervention once weekly for 15 weeks. A matched, non-exercising control group (CG) consisted of 30 physically inactive adults (80% female, 45 ± 11 years). PL, compliance and sociodemographic parameters were measured. PL was evaluated by a questionnaire, covering five domains: physical activity behaviour, attitude towards a physically active lifestyle, exercise motivation, knowledge and self-confidence/self-efficacy. Data were analysed using ANCOVA models, adjusted for age, gender and BMI at baseline.

**Results:**

At post-training intervention, the IG showed significant improvements in PL (*p* = 0.001) and in the domains physical activity behaviour (*p* = 0.02) and exercise self-confidence/self-efficacy (p = 0.001), with no changes overserved for the CG regarding PL and those domains. No intervention effect were found for the other three domains, i.e. attitude, knowledge and motivation. Additionally, for the IG baseline BMI was identified to be positively correlated with physical exercise-induced improvements in PL (β = 0.51, *p* = 0.01).

**Conclusions:**

The results from this study are very useful for further public health activities, which aim at helping physically inactive adults to adopt a physically active lifestyle as well as for the development of further PL intervention strategies. This pilot-study was a first attempt to measure PL in inactive adults. Yet, a validated measurement tool is still not available. Further research is necessary to determine the psychometric properties for this PL questionnaire.

**Trial registration:**

German Clinical Trials Register (DRKS), DRKS00013991, date of registration: 09.02.2018, retrospectively registered.

**Electronic supplementary material:**

The online version of this article (10.1186/s12889-019-6719-z) contains supplementary material, which is available to authorized users.

## Background

Over the last decades prevalence of overweight and obesity increased dramatically and poses a serious public health concern [1–3]. Worldwide, 10.8% in men and 14.9% in women were obese in 2014 [1]. Causes for obesity are multifactorial, including for example increasing age or a low education level [4, 5]. Above all, physical inactivity is well known as one of the key drivers for the increase in prevalence of obesity [6, 7]. Findings from a large survey of adult US-Americans highlighted that physical inactivity accounted for nearly 20% of the variance in prevalence of obesity after adjusting for age, gender, race, and median household income [7]. Recently it was shown that globally 31% of adults are physically inactive [8]. In that context, evidence highlighted the strong association between physical inactivity and premature mortality as well as a large number of non-communicable diseases (NCD), including type 2 diabetes, coronary heart disease, breast- and colon cancer [9].

Despite highly variable definitions of physical literacy (PL) [10], Whitehead’s definition of PL is widely accepted [11, 12]. She describes PL as a holistic and multidimensional construct, wherein a physically literate person has the motivation, confidence, physical competence, knowledge and understanding to value and take responsibility for maintaining purposeful physical pursuits and activities throughout the life course [13]. In general, PL is considered as a person’s capacity and commitment to a physically active lifestyle [14].

In recent years, there has been a growing interest in PL, when facing challenges of the high prevalence of physically inactivity [14, 15]. Indeed, those who are physically literate engage in regular physical activities, meet recommended physical activity guidelines, and present only a limited sedentary behaviour. In short, physical literate people follow a physical active lifestyle in a sustainable manner [16]. Therefore, enhancing individuals’ PL can be considered as a key opportunity in supporting a greater and persisting engagement in physical activities and, thereby, in preventing overweight and obesity.

Research in PL has mainly focused on children and adolescents and was, for this reason, predominately covered by physical education [11, 12]. Contrarily, some researchers pointed out that PL is not exclusively linked to physical education and recommended to foster PL even beyond schooling in adulthood and retirement [13, 17]. They emphasised that all individuals are able to get age-independently physically literate. In fact, PL can be envisioned as a lifelong journey rather than a certain end point, once reached persisting for the rest of one’s life [13]. Most significantly, however, people classified as physically inactive are known to have little progress and many setbacks along their PL journey. Such an inconsistent PL journey, in turn, results in a low PL level [17]. Thus, there is a need to encourage and support those people to ameliorate their journey in order to enhance their PL [13].

It was emphasised that physical literacy can be developed through physical activity per se [11]. Indeed, studies including physically inactive adults demonstrated a clear impact of physical exercise training on selected PL domains, such as physical activity behaviour [18] and exercise self-confidence/self-confidence [19, 20]. However, when considering PL as a multifaceted construct of physical, affective and cognitive domains, an exercise training alone appears not be sufficient to gain improvements in all domains. In particular, such unidirectional approach might be a less successful intervention for cognitive PL domains such as exercise knowledge. Consequently, when exercise training is applied in order to ameliorate PL from a holistic point of view, it must incorporate training elements targeting physical, behavioural, affective as well as cognitive elements of physical activity simultaneously. Yet, to the best of our knowledge, no attention has been paid to a physical exercise intervention based on such a holistic approach so far, when dealing with promoting strategies of PL. Hence, the primary aim of this pilot-study was to assess the effects of a holistic physical exercise training on PL among physically inactive adults. We hypothesised that physically inactive adults would benefit from such a holistic training program, regardless of the applied mode of training, but considering physical, behavioural, affective and cognitive elements of physical activity. A second aim of this pilot study was to identify training- and sociodemographic parameters affecting changes in PL.

## Methods

### Study design

This pilot-study was a non-randomised controlled trial with outcome assessements at baseline and after a exercise training program. Participants within the intervention group (IG) received a holistic physical exercise training, while the selected control group (CG) did not. IG and CG were matched for gender, anthropometric parameters and education levels.

### Consent procedures

All participants gave their written informed consent before any trial-related activities. Ethical approval, in accordance with the Helsinki Declaration, was sought and obtained from the Research Ethics Committee of the University of Graz, Styria, Austria (Number 62–2015/16).

### Study participants

Inclusion criteria were (i) “physically inactive”, whereby we defined “physical inactivity” twofold, either by not meeting the American College of Sport Medicine’s (ACSM)/American Heart Association’s (AHA) physical activity recommendation for healthy adults [21] or by not meeting the scoring criteria of “minimally active” in the International Physical Activity Questionnaire – Short Form (IPAQ-SF) [22] (Table 1), (ii) age ranging between 18 and 65 (both inclusive) and (iii) the physical and mental ability to participate in the exercise training as assessed by a physician. Exclusion criteria was any evidence of an acute or chronic disease, which prevented a safe participation in the trial-related intervention.

**Table 1:** Assessment criteria for physically inactive

IPAQ-SF	ACSM/AHA
Three or more days of vigorous activity of at least 20 min per day, **OR**	Moderate-intensity aerobic (endurance) physical activity for a minimum of 30 min on 5 days each week^a^, **OR**
Five or more days of moderate-intensity activity or walking of at least 30 min per day, **OR**	Vigorous-intensity aerobic activity for a minimum of 20 min on 3 days each week^b^, **OR**
Five or more days of any combination of walking, moderate-intensity or vigorous intensity activities achieving a minimum of at least 600 MET-minutes per week	Combinations of moderate- and vigorous-intensity activity, which meet the recommendation mentioned above*, **AND**
	At least twice each week strength-based activities using the major muscles of the body in order to maintain or increase muscular strength and endurance**

*IPAQ-SF* International Physical Activity Questionnaire – Short Form, *ACSM/AHA* American College of Sports Medicine / American Heart Association, *HEPA active*, health enhancing physical activity active

aAerobic training related aspects

bStrength training related aspects

### Study procedures

**Fig. 1:**
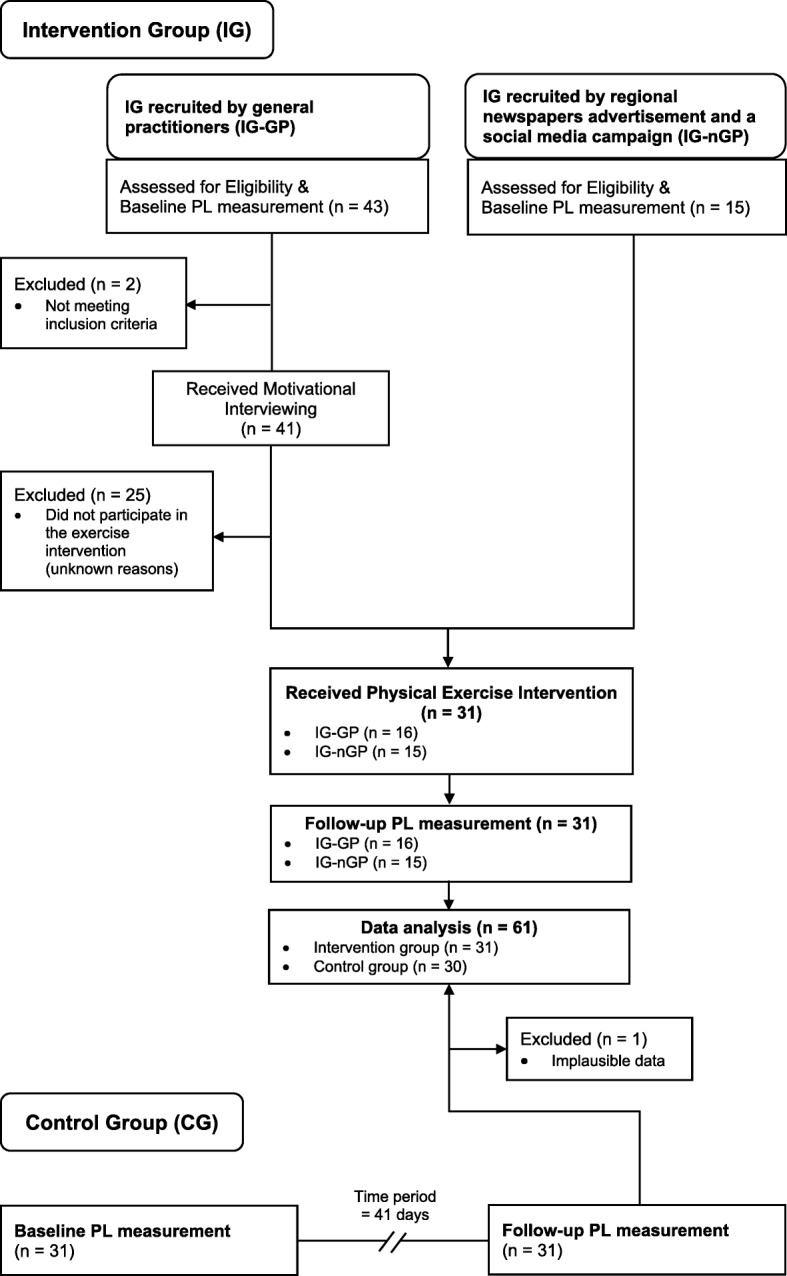
Flow diagram of recruitment numbers. *PL*: Physical Literacy

Recruitment for the IG of this pilot-study was conducted from 6th of October 2016 to 10th of December 2016, while the intervention began already on 12th of October 2016 and lasted until 1st of February 2017. As outlined in Fig. 1, IG participants were recruited by six general practitioners (subsample referred as IG-GP) as well as by regional newspapers advertisement and a social media campaign (IG-nGP). Concerning the IG-GP recruitment, baseline measurement of PL was conducted while the patients were in the practitioners’ waiting room, after signing the informed consent. Eligible participants were motivated by the general practitioners via motivational interviewing to attend the training program. Motivational interviewing was defined as “directive, client-centred counselling style for eliciting behaviour change by helping clients to explore and resolve ambivalence” [23]. Only one short 2-min session of motivation interviewing was delivered, in order to favour the participation of eligible people in the intervention. With regard to the IG-nGP recruitment, screening for eligibility and the baseline measurement of PL were conducted immediately prior to the first training sessions of those people. Additionally, those IG-nGP participants, who were not recruited by a general practitioner, were asked to consult their general practitioner to assess their physical and mental ability to participate in the physical exercise intervention, with no motivation interviewing session provided.

Matched CG was recruited by study staff after the end of the intervention period between 1st and 5th of February 2017. The time-period between baseline and follow-up measurements of PL in the CG was equivalent to the average attended training period over all participants in the IG (i.e. after 41 days). This study was performed at the FH JOANNEUM Bad Gleichenberg - University of Applied Sciences (Styria, Austria).

## Measurements

### Physical literacy

PL was considered as a holistic construct consisting of five inter-connected domains: (i) physical activity behaviour, (ii) attitude towards a physically active lifestyle (iii) exercise motivation as well as (iv) exercise knowledge and (v) exercise self-confidence/self-efficacy [13]. To the best of our knowledge, a valid measurement tool to assess PL in this multidimensional manner is only evident for children. The Canadian Assessment of Physical Literacy (CAPL) was designed to measure Physical Literacy in Canadian School children in Grades 4 to 6 [14, 15, 24]. Previous research using a confirmatory factor analysis supported the theoretical model of the CAPL and demonstrated acceptable convergent validity of this questionnaire, with comparing CAPL domain (R^2^ = 0.03–0.42) and total scores (R^2^ = 0.22) with teacher ratings [15]. No measures for reliability were provided. Consequently and corresponding with the conception of this establishes measurement tool, we combined questionnaires and questionnaire subscales to a single holistic tool, covering these five domains of PL, in order to measure PL in our pilot-study. In total, the new questionnaire consisted of 80 items. In our pilot-study the questionnaire was provided in German language. The English version of the questionnaire can be found as an additional material of this article (see Additional file 1).

Physical activity behaviour (inclusive training sessions) was measured using the IPAQ-SF with seven questions (concurrent validity: Spearman’s ρ = 0.67; test-retest reliability: ρ = 0.76) [22] and additionally with five self-constructed questions based on the ACSM/AHA physical activity guidelines for healthy adults, whereby we adapted the guideline-statements into questions [21] (i.e. 12 items in total for this subscale). An English translation of these self-constructed items can be found as an additional material of this article (see Additional file 1 – A2). This dual measurement approach of physical activity behaviour was included in our study in order to get a more comprehensive assessment of participants’ physical activity behaviour. In fact, while the IPAQ-SF encompass all daily-life physical activities, the ACSM/AHA guidelines focus solely on purposeful sport activities, including aerobic as well as also strength training related aspects of such sport activities. Based on the IPAQ-SF, participants were classified into three different activity-levels: “inactive”, “active” or “health-enhancing physical activity active (HEPA active)”. By means of ACSM/AHA guidelines (Table 1), participants were scored as “inactive” if they neither met the aerobic training (e.g. moderate-intensity aerobic physical activity of at least 30 min on 5 days each week) nor the strength training related aspects (strength-related activities on at least 2 days a week) of the recommendation. If only the aerobic training related aspects were fulfilled participants were classified as partly active and if both the aerobic training as well as the strength training related aspects of the recommendation were met participants were classified as active. This classification was different to the inclusion criteria, whereby a dichotomous approach was chosen.

The domain attitude towards a physically active lifestyle were assessed with four items based on the Stanford Five City Study questionnaire (internal consistency: α = 0.65; [25, 26]). Moreover, six additional items were constructed for this pilot-study specifically in order to get a more detailed assessment of this domain. In total, 10 items covered the domain attitude. An English translation of the six self-constructed items can be found as an additional material of this article (see Additional file 1 – C).

Exercise motivation was assessed by the Sport Motivation Scale (SMS28) amotivation subscale [27], the Situational Motivation Scale (SIMS) amotivation subscale ([28] and the Behavioural Regulation in Exercise Questionnaire-2 (BREQ-2) amotivation subscale [18]. An acceptable to good level of construct validly and reliability (Cronbach’s Alpha) was reported from previous studies for all three subscales (SMS28, [27]; SIMS, [28]; BREQ-2, [29, 30]). These questionnaire subscales were merged to one exercise motivation scale (12 items). Exercise self-confidence/self-efficacy was measured using the Body Image Questionnaire (FKB-20) [31] and four subscales regarding exercise self-efficacy, which were extracted from the research project “Risk Appraisal Consequences in Korea” (RACK) [32] (41 items in total). Regarding the FKB-20, two inverse-poled items (item 5 and 19) were reformulated in order to reverse the polarity of them, as recommended by Albani et al. [31] for forthcoming applications of this questionnaire. Acceptable to good construct and criterion validly (respectively) and reliability (Cronbach’s Alpha) is evident for the FKB-20 [31, 33] as well as for the four exercise self-confidence/self-efficacy subscales [34, 35]. All items of the domains motivation, attitude and self-confidence/self-efficacy were answered on a 5-point-Likert-type-scale.

The domain exercise knowledge was assessed with five self-constructed open-ended items, based on previous study by Fitzgerald et al. [26]. Four questions were derived from the ACSM/AHA physical activity recommendations for healthy adults [21] and one question concerned a person’s target heart rate when exercising with moderate intensity. The reason to add the question about target heart rate was that the majority of people have difficulties in understanding exercise intensities, which eliciting health benefits. In particular the intensity of moderate physical activities is often underestimated [36]. All answers referring these five open-questions were coded dichotomously as correct or incorrect. An English translation of all knowledge items can be found as an additional material of this article (see Additional file 1 – D).

In correspondence with the scoring procedure of the CAPL [15], a composite score for each of the five domains (ranged between 0 and 100%) and total score for the whole PL questionnaire, which also ranged between 0 and 100%, were calculated. The total score of the questionnaire was a composite calculation in which each of the five domains was weighted equally with 20%. A higher score indicated a greater PL. In this pilot-study only a single method instrument (i.e. self-report) was applied, whereas the CAPL used objective and self-reported measurements with more weighting given to objective measurements (i.e. pedometers). Due to no prior knowledge for weighting in the scoring procedure (e.g. Delphi-process), all domains were equally weighted.

The domain physical activity behaviour was scored as shown in Table 2, by assigning a score between 0 and 100 points to each possible combination of the IPAQ-SF and the ACSM/AHA physical activity status (considering both the aerobic- and strength-related aspects of the ACSM/AHA recommendation, Table 1). The other four domains were scored in a common way by computing the mean of the item-answers for each domain separately. The percentage of the mean value in relation to the maximal possible mean score (= 5 points for knowledge and 4 points for the other domains respectively) were calculated afterwards, in order to create total domain scores. A higher percent value indicated a greater domain proficiency.

**Table 2:** Scoring of the physical literacy questionnaire – domain physical activity behaviour

IPAQ-SF	ACSM/AHA	Domain-Score
Inactive	Inactive	0%
Inactive	Partly active (only aerobic aspects met)	25%
Active	Inactive	25%
Active	Partly active (only aerobic aspects met)	50%
HEPA active	Inactive	50%
Inactive	Active (both aerobic and strength aspects met)	50%
Active	Active (both aerobic and strength aspects met)	75%
HEPA active	Partly active (only aerobic aspects met)	75%
HEPA active	Active (both aerobic and strength aspects met)	100%

*IPAQ-SF* International Physical Activity Questionnaire – Short Form, *ACSM/AHA* American College of Sports Medicine / American Heart Association, *HEPA active* health enhancing physical activity active

### Sociodemographic and antropometric data

Questions regarding age, weight, height, acute and chronic diseases and education-level were included in our PL questionnaire. Weight and height was used to calculate the BMI of each participant (kg/m^2^).

### Holistic phyiscal exercise intervention

During the 15-weeks-intervention period, IG participants attended a holistic physical exercise training once weekly (15 training days in total), designed to improve physical, behavioural, affective and cognitive elements of physical activity. The intervention was supervised by an experienced sport scientist. Compliance is a challenge in all intervention studies including physically inactive people [37]. However, providing participants more options in terms of the training mode or in the time regime of training, it is posited to positively influence compliance in such a sample [38, 39]. To address this issue, all training days consisted of three 50-min-sessions, focusing either on strength-, endurance- or on multimodal (combination of strength- and endurance) related activities, whereby participants in our pilot-study were allowed to attend one, two or all three sessions per training day (according to their preferences). All three sessions had the same structure: each session began with a warm-up and mobilisation phase followed by conditioning phases involving session specific exercises mixed with a physical activity knowledge transfer (e.g. activity games, which were designed to mediate cognitive elements of exercising and physical activity, respectively). The strength sessions involved strength-related activities such as dynamic exercises for the whole body with Thera-Bands®, Gymsticks®, Pezzi balls® and Mini-Bands® or simple bodyweight exercises. After every five exercises, there was a recovery period of at least 3 min (usually four periods per session), in which the sport scientist provided and discussed information on, e.g., a health oriented resistance training or the physical and mental benefits of a physically active lifestyle. Moreover, the sport scientist explained how they affect an individual’s body and health, during participants performed demonstrated exercises. The conditioning phase of the second (i.e. endurance) session was separated into two parts, including both endurance related activities. The first part included a running/walking game, which were designed to mediate cognitive elements of exercising and physical activity, respectively (one game was delivered per session; an example can be found as an additional material of this article (see Additional file 2). The second part involved step-aerobic or other different kinds of walking/running games. The conditioning phase of the third session (i.e. multimodal) included tools from the first and second session in an equal manner, usually ten strength-related exercises (with two recovery periods) and one running/walking games targeting on a physical activity knowledge transfer. For all sessions, in order to promote *mastery experiences*, one of the strongest source of self-efficacy [40, 41], all exercises and games were tailored to o the physical and mental competences of the participants. To give one example, steps were, if necessary, imitated with a tape on the ground, once step aerobics was applied, to make sure, that each participant could successfully master the choreography. Additionally, as a further source of self-efficacy [40, 41], participants were encouraged, by giving positive feedback such as praising their good past performances and efforts after each session. Lastly, at the end of every training day, participants received print materials such as fact-sheets and folders, which outlined the national physical activity guidelines [42], simple ways to fit more physical activity into a busy day or the health risks of a physically inactive lifestyle.

To determine exercise intensity, participants were asked to wear a heart rate-monitor (Polar 610 and 810, Polar Electro OY, Kempele, Finland) during the sessions and to exercise within their moderate-intensity heart rate zone. Moderate intensity was set between 55 and 69% of a person’s maximum heart rate (HR_max_) according to ACSM [43], whereby a person’s HR_max_ was calculated using the age-predicted maximum heart equation of Tanaka et al. [HR_max_ = 208 – (0,7 * age)] [44]. Moreover, at the end of each session participants were asked to fill in their rate of perceived exertion on a Borg-Scale [45].

### Statistical analysis

All continuous variables were expressed as mean ± standard deviation and categorical variables as frequency (%; unless otherwise stated). The Shapiro-Wilk normality test was performed to assess the assumption of normal data distribution. Baseline data were analysed by unpaired t-test, Mann–Whitney U-test and χ^2^-test or Fisher’s exact test for categorical variables. Cronbach’s alpha coefficient (α) for internal consistency was used to test for reliability, with α ≥ 0.7 indicating acceptable, α ≥ 0.8 indicating good, and α ≥ 0.9 indicating very good internal consistency.

A two-way ANCOVA for repeated measures using age, gender and baseline BMI values as covariates (rmANCOVA, 2 × 2 design) was applied to detect changes of PL in IG and CG over time (baseline compared to follow-up). Factors were time (baseline to follow-up) and group (IG to CG). Paired t-tests with Bonferroni correction (set at a significance level of *p* < 0.025) within IG and CG were utilised as post-hoc-analysis. Similarly, changes in self-confidence/self-efficacy were analysed by rmANCOVA using Bonferroni’s correction as a post-hoc analysis. Given that assumption for parametric rmANCOVA models were not met for the other four domains (physical activity behaviour, attitude, motivation, and knowledge), nonparametric rmANCOVA applying the Puri and Sen L statistics for ranked data were conducted [46, 47]. The statistical analysis is based on rank-transformations of observed values, whereby conventional statistical analysis was run on these ranked values subsequently. For post hoc analysis, Wilcoxon signed-rank tests with *p*-value adjustment (p < 0.025) were performed. These non-parametric procedures have also been used recently in physical exercise intervention studies (e.g. [48, 49]). Moreover, in order to reveal the effect of the two different recruiting ways of the IG (IG-GP vs. IG-nGP), subsamples analyses were performed on PL and all domain scores. Thereby, two rmANCOVA (2 × 2 design) were applied to compare both the CG and IG-GP subsample and the CG and IG-nGP subsample over time (baseline compared to follow-up). To estimate rmANCOVA effect sizes, partial eta squared (η_p_^2^) were additionally computed with η_p_^2^ ≥ 0.01 indicating small, η_p_^2^ ≥ 0.059 medium and η_p_^2^ ≥ 0.14 large effects.

In order to reveal factors influencing exercise-induced improvements in PL, a stepwise linear regression analysis using the delta values of IG participants’ PL (PL follow-up minus PL baseline) as the dependent variables was carried out. Age, gender, baseline and follow-up BMI, changes in BMI (baseline BMI minus follow-up BMI values), different education levels, chronical diseases (yes/no) and exercise parameters including compliance, performed total training sessions and training minutes as well as exercise intensity were introduced as independent variables in the stepwise procedure. The regression analysis was adjusted for age, gender and baseline BMI values.

Statistical analysis was performed using the SPSS software (version 24.0), and *p* < 0.05 was considered statistically significant. Post hoc power analysis was carried out applying G*Power (version 3.1) [50].

## Results

Forty-one eligible people were motivated via motivational interviewing by general practitioners to participate (Fig. 1). Twenty-five people withdrew for unknown reasons before starting the training intervention. In total, 31 physically adults were allocated to the IG (16 IG-GP and 15 IG-nGP) and completed both the baseline and follow-up measurement of PL. Thirty-one physically inactive adults were included in the selected CG. Due to plausibility check one person was excluded within the CG (implausible data in the domain physical activity behaviour at follow-up measurement). Consequently, data of 30 CG participants were analysed.

### Baseline characteristics

Participant characteristics were similar for IG and CG (*p* ≥ 0.05; Table 3). Furthermore, no differences were found in the total PL score or in any domain scores of the PL model between both groups at baseline. A Table outlining the descriptive characteristics of the IG subsamples (IG-GP and IG-nGP) together with data of the CG can be found as supplement material of this article (see Additional file 3). The IG-GP subsample had significant higher values regarding initial weight, BMI and number of participants suffering from chronic diseases when comparing with the IG-nGP subsample (all *p* ≤ 0.01) and CG (all p ≤ 0.01), respectively. IG-nGP participants were significant younger than those in the IG-GP (*p* < 0.001) and CG (*p* < 0.006). Additionally, they presented higher scores for total PL (*p* = 0.009) and the domain self-confidence/self-efficacy (*p* = 0.005) in comparisons to the IG-GP participants.

**Table 3:** Participant 913 characteristics of IG and CG

Variables	IG	CG	p-value
Gender (females)	25 (81%)	24 (80%)	0.95
Age (years)	44 ± 16	45 ± 11	0.98
Anthropometry			
Height (cm)	168 ± 7	169 ± 7	0.62
Weight (kg)	75 ± 22	72 ± 15	0.80
BMI (kg/m^2^)	27 ± 8	25 ± 4	0.79
Education level			
Compulsory school (n)	5 (16%)	3 (10%)	0.57
Apprenticeship/Professional school degree (n)	12 (39%)	15 (50%)	
School leaving examination (A-Level) (n)	12 (39%)	8 (27%)	
Grad/professional degree (n)	2 (6%)	4 (13%)	
Chronic diseases (n)	12 (39%)	6 (20%)	0.13
Physical Literacy (%)	59 ± 11	59 ± 10	0.75
Physical activity behaviour (%)^a^	25 (25)	25 (25)	0.82
Attitude (%)	63 ± 18	62 ± 17	0.86
Motivation (%)	78 ± 17	74 ± 14	0.25
Knowledge (%)	55 ± 16	57 ± 22	0.43
Self-confidence/Self-efficacy (%)	68 ± 13	66 ± 11	0.56

Values are given as mean ± SD or as frequency (%); *IG* Intervention group, *CG* Control group, *BMI* body mass index;

^a^Given as median ± IQR, because ordinal scaled

### Compliance and training parameters

Due to the time overlapping situation between the recruitment and the intervention period, the average potential training period was 12.5 ± 3.4 training days. Mean compliance with the holistic physical exercise training was 48 ± 29%. Interestingly, subsample analysis showed that IG-GP participants had a higher compliance than those in the IG-nGP (60 ± 25% vs. 35 ± 27%, *p* = 0.01). Exercise characteristics of IG and IG subsamples are outlined in Table 4. Overall and explicitly for the multimodal training, mean performed training sessions and training time training were significantly higher for the IG-GP subsample compared to the IG-nGP. Besides, for the multimodal training IG-GP participants showed higher values on the Borg-Scale in comparison to those in the IG-nGP. Fourteen IG participants (45%) attended 3.1 ± 2.2 times both the strength-based and the endurance-based training at the same day. One adverse event (dizziness) was registered during the mobilisation part of an endurance-based session.

**Table 4:** Exercise characteristics o 918 f IG and IG subsamples

Variables	IG	Subsamples	
		IG-GP	IG-nGP
Training sessions (n)^a^	7.3 ± 5.0	8.7 ± 4.3^*^	5.7 ± 5.3
Strength	5.0 ± 3.0	5.6 ± 3.1	4.2 ± 2.9
Endurance	3.5 ± 3.0	3.4 ± 3.2	3.7 ± 2.5
Multimodal	4.1 ± 3.4	8.4 ± 4.3^**^	2.0 ± 1.7
Training time (min)^a^	352 ± 242	428 ± 208^*^	272 ± 257
Strength	232 ± 151	273 ± 152	186 ± 144
Endurance	176 ± 151	171 ± 170	183 ± 128
Multimodal	203 ± 159	418 ± 215^**^	100 ± 83
% of predicted HF_max_^a, b^	67 ± 9	69 ± 10	64 ± 6
Strength^b^	65 ± 9	67 ± 11	62 ± 3
Endurance^b^	69 ± 9	71 ± 10	65 ± 8
Multimodal^b^	68 ± 10	70 ± 12	64 ± 6
Borg scale^a, c^	12 ± 2	12 ± 1	12 ± 2
Strength^c^	13 ± 1	13 ± 1	13 ± 1
Endurance^c^	12 ± 2	12 ± 2	13 ± 2
Multimodal^c^	11 ± 2	12 ± 1*	10 ± 2

Values are given as mean ± SD; *IG* intervention group, *IG-GP* IG participants, recruited from GP, *IG-nGP* IG participants not recruited from GP, *HFmax* maximum heart rate; ^*^p < 0.05, different from IG-nGP^; **^p < 0.01, different from IG-nGP

^a^Without considering different modes of training

^b^All values indicating “moderate” intensity according to the ACSM [41], with the exception of the endurance and multimodal training in the IG-GP subsample, which demonstrate “vigorous” intensity

^c^All values indicating “moderate” intensity according to the ACSM [41], with the exception of the multimodal training in the IG and in the IG-GP subsample, which demonstrate “light” intensity

### Reliability of the physical literacy (PL) questionnaire

Taking the data from IG and CG participants (*n* = 61) together, the Cronbach’s α coefficients (baseline/follow-up) were α = 0.81/0.80 for the domain attitude, α = 0.86/0.81 for the domain motivation and α = 0.91/0.95 for the domain exercise self-confidence/self-efficacy. Cronbach’s α coefficients were not calculated for the domains physical activity behaviour and knowledge, because these domains are represented by causal or composite items, which independently contribute to the domain scores. The assessment of internal structure (e.g., using Cronbach’s alpha) is only relevant for constructs (i.e. domains) consisting of reflective items [51]. The test-retest procedure would have been possible for those two domains, yet it was not applied due to the fact, that the results of this estimation would have been biased by the training intervention conducted between the two measurements points.

### Total physical literacy (PL) score

**Fig. 2:**
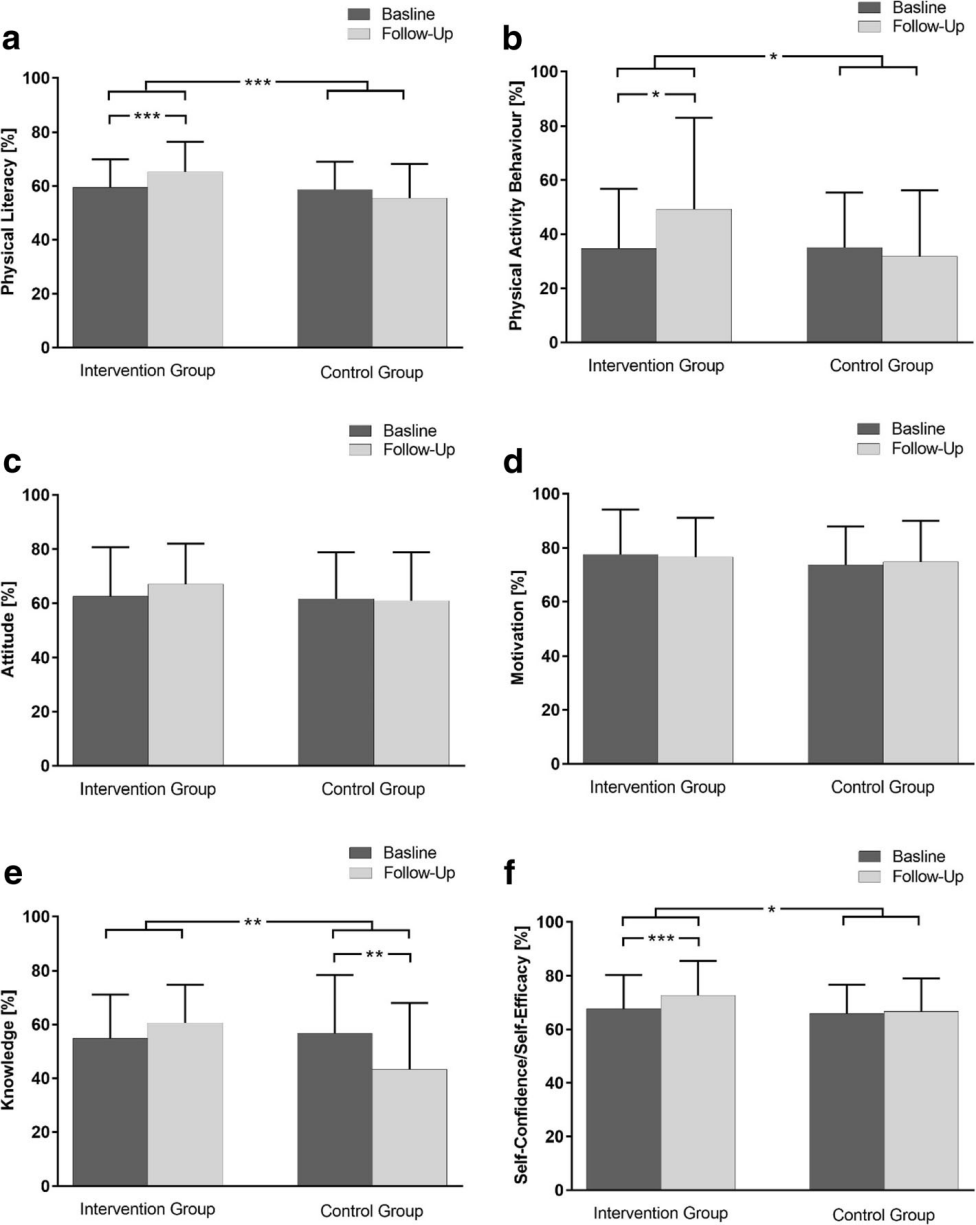
Physical literacy (PL) (**a**) and all five domains of PL across the intervention period (**b** = Physical Activity Behaviour, **c** = Attitude, **d** = Motivation, **e** = Knowledge, **f** = Self-Confidence/Self-Efficacy). Baseline: charcoal grey; follow-up: light grey; *IG*: intervention group; *CG*: control group. Values are given as mean ± SD, for a better clarity physical activity behaviour (ordinal scaled) is also given as mean ± SD. ^*^p < 0.05; ^**^p < 0.01; ^***^p < 0.001

For the total PL score, a significant time × group interaction (*p* < 0.001) with a large effect size (η_p_^2^ = 0.21) and a statistical power of > 0.99 according to a post hoc power analysis was evident. Post-hoc testing indicated that PL increased on average by ≈ 12% in the IG at follow-up (59 ± 11 vs. 65 ± 11%, p < 0.001), whereas in the CG PL remained unchanged (59 ± 10 vs. 56 ± 13%, *p* = 0.04; Fig. 2a). A subsample analysis revealed significant time × group interaction effects, when both the IG-GP and CG (p = 0.04, η_p_^2^ = 0.18) and IG-nGP and CG (*p* = 0.03, η_p_^2^ = 0.12) were compared. Post hoc tests demonstrated a significant increase in PL at follow-up in comparison to the baseline for the IG-GP (55 ± 10 vs. 62 ± 9%, *p* = 0.02) and the IG-nGP (64 ± 9 vs. 69 ± 13%, p = 0.02).

### Domain physical activity behaviour

Physical activity behaviour revealed a significant interaction for changes when comparing IG and CG (p = 0.04) with a medium effect size (η_p_^2^ = 0.07). Post hoc analysis showed that IG had a significantly higher domain score for physical activity behaviour at follow-up in comparison to the baseline (Median (IQR)) (25 (25) vs. 50 (50)%, p = 0.02), while no changes were observed for the CG (25 (25) vs. 25 (50)%, *p* = 0.26; Fig. 2b). In a subsample analysis no significant time × group interaction effects were evident, when both the IG-GP and CG (*p* = 0.56, η_p_^2^ < 0.01) and IG-nGP and CG (*p* = 0.06, η_p_^2^ = 0.09) were compared (IG-GP: 25 (25) vs. 25 (50)%; IG-nGP: 25 (25) vs. 50 (50)%).

### Domain attitude

Neither a significant time effect (*p* = 0.67, η^2^ = 0.01) nor a significant time × group interaction (*p* = 0.14, η_p_^2^ < 0.01) occurred for the domain attitude towards a physically active lifestyle (IG: 63 ± 18 vs. 67 ± 15%; CG: 62 ± 17 vs. 61 ± 18%; Fig. 2c). In a subsample analysis results were similar, when both the IG-GP and CG (time × group interaction: *p* = 0.15, η_p_^2^ = 0.05; time effect: *p* = 0.48, η_p_^2^ < 0.01) and IG-nGP and CG (time × group interaction: *p* = 0.55, η_p_^2^ < 0.01; time effect: p = 0.06, η_p_^2^ < 0.09) were compared (IG-GP: 59 ± 18 vs. 65 ± 12%; IG-nGP: 67 ± 18 vs. 69 ± 18%).

### Domain motivation

Neither a significant main effect for time (*p* = 0.70, η_p_^2^ < 0.01) nor a significant time × group interaction (*p* = 0.52, η_p_^2^ = 0.01) was observed in exercise motivation (IG: 78 ± 17 vs. 77 ± 15%; CG: 74 ± 14 vs. 75 ± 15%; Fig. 2d). In a subsample analysis results remained non-significant, when both the IG-GP and CG (time × group interaction: *p* = 0.77, η_p_^2^ < 0.01; time effect: *p* = 0.61, η_p_^2^ < 0.01) and IG-nGP and CG (time × group interaction: *p* = 0.23, η_p_^2^ = 0.03; time effect: *p* = 0.16, η_p_^2^ = 0.05) were compared (IG-GP: 74 ± 14 vs. 74 ± 15%; IG-nGP: 82 ± 19 vs. 79 ± 15%).

### Domain knowledge

A significant interaction effect (*p* = 0.004) with a medium to large effect size (η_p_^2^ = 0.13) for changes for the domain exercise knowledge was evident when IG and CG were compared. However, subsequent post hoc analysis indicated that the domain score for knowledge declined significantly in the CG (57 ± 22 vs. 43 ± 25%, p = 0.004), whereas IG showed no significant changes in comparison to the baseline (55 ± 16 vs. 61 ± 14%, = 0.11) (Fig. 2e). Subsample analysis showed a significant time × group interaction effect, when comparing IG-nGP and CG (*p* = 0.03, η_p_^2^ = 0.11). Yet, post hoc tests demonstrated no significant changes for IG-nGP in exercise knowledge at follow up in comparison to the baseline (56 ± 11 vs. 63 ± 15%, *p* = 0.10). No significant time × group interaction effect was evident when the IG-GP subsample and CG were compared (p = 0.10, η_p_^2^ = 0.06; IG-GP baseline-follow-up: 54 ± 20 vs. 59 ± 14%).

### Domain self-confidence/self-efficacy

Regarding the domain score for self-confidence/self-efficacy a significant time × group interaction (*p* = 0.04) with a medium effect size (η_p_^2^ = 0.08) was evident. Subsequent post-hoc testing indicated an increase by ≈ 9% in the IG (68 ± 13 vs. 73 ± 13%, *p* = 0.001), whereas no changes were observed in the CG in comparison to the (66 ± 11 vs. 67 ± 12%, *p* = 0.56; Fig. 2f). A subsample analysis revealed a significant time × group interaction, when IG-GP and CG were compared (*p* = 0.01, η_p_^2^ = 0.16) with post hoc showing a significant increase for IG-GP at follow up in comparison to the baseline (62 ± 13 vs. 69 ± 13%, *p* = 0.003). No significant time × group interaction effect was observed when IG-nGP subsample and CG were compared (*p* = 0.93, η_p_^2^ < 0.01; IG-nGP baseline-follow-up: 74 ± 9 vs. 77 ± 12%).

### Factors influencing exercise-induced improvements in PL

In a stepwise linear regression analysis adjusted for age and gender (R^2^ = 0.25), only baseline BMI (β = 0.51, p = 0.01) was positively associated with exercise-induced improvements in PL. Due to the significance of baseline BMI in the stepwise procedure, no adjustment was carried out for this variable. Neither age, gender, delta BMI, different education levels, chronical diseases, recruitment way of IG nor exercise parameters including compliance, performed total training sessions and training minutes as well as exercise intensity were identified as explanatory variables of changes in PL.

## Discussion

This pilot-study is the first study assessing effects of a holistic physical exercise training on PL among physically inactive adults, whereby PL is considered as a multifaceted construct of physical, affective and cognitive domains. The main result of the present study indicated that participating in a holistic exercise training ameliorates total PL score and selective PL domains such as physical activity behaviour and exercise self-confidence/self-efficacy among physically inactive adults. Additionally, we found that improvements of PL are positively correlated with BMI values at baseline. However, when interpreting these findings, we need to consider that this pilot-study was a first attempt to measure PL with a non-validated measurement tool in physically inactive adults. Therefore, drawing conclusions based on our findings should be done with caution.

Even though we have applied established approaches to improve compliance (see methods section), the mean compliance in our study was quite low (48 ± 29%). We suggest that the low compliance may have been due to limited experience with exercise training, conducting the training intervention in autumn and winter time (higher prevalence of illnesses), difficulties in prioritising time for exercise or to symptoms in relation to chronical diseases (e.g. tiredness), as nearly 40% of our IG participants reported to suffer from at least one chronic disease. Nevertheless, our results show that IG subsample recruited by general practitioners (IG-GP) had a higher compliance to our intervention than that, who were recruited by regional newspaper advertisement and a social media campaign (IG-nGP). The positive effects of motivational interviewing in order to increase individuals compliance are well-established [52]. However, whether it was the motivational interviewing, or the general practitioners included in the recruitment process themselves who facilitated of the positive effect on PL cannot be distinguished. Since general practitioners are held in high reputation in Austria [53, 54], is it likely that general practitioners’ advice has more influence on a person’s conscientiousness in terms of initiating and maintaining an action than those from other people.

### Physical literacy

Evidence has highlighted that all individuals can make age-independent progress on their PL journey to become physically literate [13, 17]. However, supporting strategies of PL have mainly targeted children and adolescents [11, 12]. The majority of participants in the present study were physically inactive and overweight midlife-females, who showed clear physical exercise-induced improvements in PL. In particular, those participants in the IG-GP subsample, who were older, had a higher prevalence of chronical diseases and BMI values as well as lower baseline values in total PL and in the domain self-confidence/self-efficacy demonstrated a larger increase in PL increase in comparison to the IG-nGP subsample. Therefore, a holistic physical exercise training might be seen as a safe and feasible intervention to help to restart a PL journey in later adulthood. PL is considered as an antecedent of physical activity [11] and physically literate persons are suggested to have an ameliorated capacity and commitment to a physically active lifestyle in a sustainable manner [14]. Against this background, our finding might be important for further public health and physical activity promoting activities in western countries [55].

The increase of the PL level was primarily explained by improvements in the domains physical activity behaviour and exercise self-confidence/self-efficacy, as no significant intervention effects for the domains exercise motivation, knowledge and attitude towards a physical active lifestyle were found. Improvements in PL were positively correlated with baseline BMI values in our study. Since it is clear that an increased BMI is inversely associated with one’s physical activity behaviour [56] and exercise self-confidence/self-efficacy [57], those with highest BMI might have benefited most from the intervention in terms of their PL level due to poorer baseline values in those domains. As aforementioned, the IG-GP subsample demonstrated higher BMI values and lower scores for total PL and domain self-confidence/self-efficacy at baseline compared to the IG-nGP subsample. For this reason a subsample analyses were performed, which revealed larger intervention effects for the total PL and the domain self-confidence/self-efficacy. However, it is important to note that IG-GP participants had a higher compliance than those in the IG-nGP subsample, which could have also mediated this finding. Further work is required to fully understand this improved response pattern of physical exercise-induced improvements in PL among people with increased BMI values.

The finding for the domain exercise self-confidence/self-efficacy is consistently in line with results from previous exercise training studies [19, 20], which found also that physical exercise leads to improvements of individuals’ exercise self-confidence/self-efficacy. However, it is worth noting that not exercise training involvement per se leads automatically to positive changes in exercise self-confidence/self-efficacy. Reasons explaining the origin of our observed improvements in this domain may be found in the holistic conception of our intervention as well as in the fact that we applied well-established techniques for promoting exercise self-efficacy in our study (see methods section). Evidence has clearly shown that implementing such techniques in training sessions lead to improvements of exercise self-efficacy [40, 41], whereby a higher exercise self-efficacy, in turn, influence individuals’ exercise self-confidence positively [58]. With regard to the subsample analysis of this domain, we found only significant improvements for the IG-GP subsample. However, while very high improvements (large effect size) were evident for the latter mentioned group, the IG-nGP participants demonstrated already a very high level of exercise self-confidence/self-efficacy at baseline. We suggest that high baseline values did not permit a further increase in the IG-nGP subsample referring to this domain.

A stronger sense of exercise self-confidence/self-efficacy is well-known as a predictor of initiating and maintaining physical activity [59, 60]. Unquestionable, our finding in terms of the domain physical activity behaviour may have important practical implications, because we found a significant intervention effect at an exercise dosage much lower than commonly used in previous exercise training studies [18].

Even though there is evidence in favour of physical exercise interventions to be beneficial for exercise motivation [61], we did not find increases in our study. This might be caused by higher baseline-values of exercise motivation in the present study in comparison to earlier studies. It is likely that participants were already well-motivated regarding physical activity at study baseline, but, for example, the absence of a local training program dedicated exclusively for physically inactive adults, might have prevented them from regular physical activity in their past.

In order to enhance exercise knowledge, we implemented exercises mixed with a physical activity knowledge transfer in the conditioning phase of all three modes of training. Nonetheless, our training program was not able to increase knowledge. It is reasonable to assume that our intervention effect on exercise knowledge was limited by the low compliance in our study in combination to the fact, that our training program took place only once a week. Moreover, we have to acknowledge that the psychometric properties of the subscale in our questionnaire might have been moderated by a limited number of items which have poor item difficulties.

In contrast to previous research [62], we did not observe an increase of physical activity attitude in the IG. In general, attitude towards a physically active lifestyle is determined by a person’s positive or negative assessment of engaging in physical activity [63]. In fact, perceiving benefits by participating in a training program (e.g. improved aerobic fitness) will lead to a more positive attitude. Since physiological and mental adaptations predominantly interact in a dose-response manner [64, 65], it might be that our comparatively short intervention period, along with a low compliance, limited the degree of perceived benefits of training among our IG participants.

Some limitations of our pilot-study are important to consider when interpreting the obtained results and the used methodological approach. First, due to the nature of a pilot-study a preceding validation study with a representative sample size was not feasible. We highly recommend conducting further studies in order to support the theoretical model of our PL questionnaire (e.g. using a confirmatory factor analysis approach) as well as studies investigating the standard psychometric properties of our questionnaire i.e. convergent and divergent validity and test-retest reliability. Moreover, during the data interpretation phase of our study, doubts have been raised whether the domain exercise knowledge, has sufficient sensitivity in order to detect changes over time (i.e. responsiveness). Additional research would be necessary to revise and extent this subscale. The second important limitation refers to the scoring procedure of our questionnaire, which was adapted from the CAPL [15]. As Corbin et al. [10] pointed out the use of a composite score has the potential to misguide, when assessing the multidimensional nature of PL. To be precise, a person with a high score on one specific PL domain (e.g. exercise knowledge) and a low score on another (e.g. physical activity behaviour) could have a similar composite score (total PL score) than a person who had reverse scores regarding these domains (i.e. low exercise knowledge and high physical activity behaviour). This may not only be seen as a limitation of our questionnaire, but also for the CAPL itself [14, 15]. Therefore, further studies should try to develop more appropriate assessments procedures, beyond the use of a composite score. Moreover, within our scoring procedure, each domain was equally weighted. Given the fact that PL is described as the antecedent of physical activity [11], one can speculated that the domain physical activity behaviour is more important within the PL model than the other domains. Indeed, the CAPL gave a higher weighting to the domain physical activity behaviour. Yet, this arose due to the fact that it was objectively and subjectively measured and rather than this domain was considered as more important than the others. Applying a Delphi process in order to collate expert opinions would help to clarify if all domains should be equally weighted when only a self-reported instrument is used.

Lastly, IG participants were allowed to join one, two or all three modes of training sessions (strength, endurance and multimodal) per intervention day. Consequently, no inter group differences regarding the different modes of training on changes in PL were investigated.

## Conclusions

In conclusion, the present study was designed to assess the effects of a holistic physical exercise training on PL among physically inactive adults. Our finding highlighted that participating in such intervention is associated with clear improvements in PL, whereby a positive correlation between changes in PL and baseline BMI values was evident. Regarding the five domains of PL, IG participants showed significant improvements in physical activity behaviour and self-confidence/self-efficacy. Yet, participants recruited by GP, who were older, had a higher BMI values and prevalence of chronical diseases, benefited more in total PL and domain exercise self-confidence/self-efficacy. No changes were found concerning the domains attitude towards a physically active lifestyle, exercise knowledge and motivation in the total sample as well as in any subsamples. Nevertheless, we suggest that IG participants were well-motivated referring to physical activity pre- and post-training intervention, but the absence of appropriate exercise facilities in their living-community might have prevented them from regular physical activity in their past. In order ensure improvements in all PL domains, further interventions should deal with a greater exercise dosage in terms of training frequency. Furthermore, we recommend an enhanced integration of general practitioners in the recruitment process of further interventions for the promotion of health and physical activity, because they may be effective to improve compliance.

Importantly, since physically literate persons are suggested to have a ameliorate capacity and sustainable commitment to a physically active lifestyle, our study results are very useful for further public health activities, which aim at helping physically inactive adults to adopt a physically active lifestyle as well as for the development of further PL intervention strategies. Overall, one should remember that this pilot-study was a first attempt to measure PL in physically inactive adults. However, a validated measurement tool is still not available and, therefore, this limitation have to be taken into account when interpreting our findings. Further research is necessary to determine the psychometric properties for this PL questionnaire.

## Additional files

Additional file 1: Physical Literacy Questionaire (English Version) (DOCX 62 kb)

Additional file 2: Running/walking - physical activity knowledge transfer (DOCX 157 kb)

Additional file 3: Descriptive characteristics of the IG subsamples (DOCX 15 kb)

## Abbreviations

ACSM/AHA: American College of Sport Medicine’s/American Heart Association’s
BMI: Body mass index
BREQ-2: Behavioural Regulation in Exercise Questionnaire-2
CAPL: Canadian Assessment of Physical Literacy
CG: Control group
FKB-20: Body Image Questionnaire
HEPA active: Health enhancing physical activity active
HR_max_: Maximum heart rate
IG: Intervention group
IG-GP: Subsample of intervention group, which was recruited by general practitioners
IG-nGP: Subsample of intervention group, which was recruited by regional newspaper advertisement and a social media campaign
IPAQ-SF: International Physical Activity Questionnaire – Short Form
NCD: Non-communicable diseases
PL: Physical literacy
rmANCOVA: two-way analysis of covariance for repeated measures
SIMS: Situational Motivation Scale
SMS28: Sport Motivation Scale
