# Supplementary material for: Holistic physical exercise training improves physical literacy among physically inactive adults: a pilot intervention study
Source: BMC Public Health. 2019 Apr 11;19:393. doi: 10.1186/s12889-019-6719-z (PMC6458734; doi:10.1186/s12889-019-6719-z)
Supplement: Supplementary file 1 — Physical Literacy Questionaire (English Version) (DOCX 62 kb) [file 12889_2019_6719_MOESM1_ESM.docx]

**Questionnaire**

Physical Literacy

Dear Participants!

We are interested in finding out your Physical Literacy level. Physical Literacy is a multidimensional construct encompassing a person’s physical activity behaviour, attitude towards a physically active lifestyle, exercise motivation, exercise knowledge and exercise self-confidence/self-efficacy.

This questionnaire was approved by the Ethics Committee of the University of Graz. The average time needed for filling in the questionnaire is 20 minutes. All answers and personal information provided will be dealt with in strict confidence and will be used only for scientific purposes. All data provided will not be disclosed to third parties.

**Personal information**

| Male O | Female O |
| --- | --- |
| Age |  |
| Weight [kg] |  |
| Height [cm] |  |

| Acute diseases | Yes O No O |
| --- | --- |
| If so, which ones? |  |
| Acute and chronic diseases | Yes O No O |
| If so, which ones? |  |

**A1: Questions related to your physical activity behaviour**

The following questions will ask you about the time you spent being physically active in the last 7 days. Please answer all questions even if you do not consider yourself an active person. Please think about the activities you do at work, as part of your house and yard work, to get from place to place, and in your leisure time for recreation, exercise or sport.

Think about all the vigorous activities that you did in the last 7 days. Vigorous physical activities refer to activities that take hard physical effort and make you breathe much harder than normal. Think only about those physical activities that you did for at least 10 minutes at a time.

| **Question 1** | **1** | **2** | **3** | | **4** | **5** | **6** | | **7** |
| --- | --- | --- | --- | --- | --- | --- | --- | --- | --- |
| During the last 7 days, on how many days did you do vigorous physical activities like heavy lifting, digging, aerobics, or fast bicycling | O | O | O | | O | O | O | | O |
|  | O No vigorous physical activities  Skip to question 5 | | | | | | | | |
| **Question 2** | **Minutes/day** | | | **Hours/day** | | | | **Don’t know** | |
| How much time did you usually spend doing vigorous physical activities on one of those days? |  | | |  | | | | O | |

Think about all the moderate activities that you did in the last 7 days. Moderate activities refer to activities that take moderate physical effort and make you breathe somewhat harder than normal. Think only about those physical activities that you did for at least 10 minutes at a time.

| **Question 3** | **1** | **2** | **3** | | **4** | **5** | **6** | | **7** |
| --- | --- | --- | --- | --- | --- | --- | --- | --- | --- |
| During the last 7 days, on how many days did you do moderate physical activities like carrying light loads, bicycling at a regular pace, or doubles tennis? Do not include walking. | O | O | O | | O | O | O | | O |
|  | O No moderate physical activities  Skip to question 5 | | | | | | | | |
| **Question 4** | **Minutes/day** | | | **Hours/day** | | | | **Don’t know** | |
| How much time did you usually spend doing moderate physical activities on one of those days? |  | | |  | | | | O | |

Think about the time you spent walking in the last 7 days. This includes at work and at home, walking to travel from place to place, and any other walking that you have done solely for recreation, sport, exercise, or leisure.

| **Question 5** | **1** | **2** | **3** | | **4** | **5** | | **6** | **7** |
| --- | --- | --- | --- | --- | --- | --- | --- | --- | --- |
| During the last 7 days, on how many days did you walk for at least 10 minutes at a time? | O | O | O | | O | O | | O | O |
|  | O No walking  Skip to question 7 | | | | | | | | |
| **Question 6** | **Minutes/day** | | | **Hours/day** | | | **Don’t know** | | |
| How much time did you usually spend walking on one of those days? |  | | |  | | | O | | |

The last question is about the time you spent sitting on weekdays during the last 7 days. Include time spent at work, at home, while doing course work and during leisure time. This may include time spent sitting at a desk, visiting friends, reading, or sitting or lying down to watch television.

| **Question 7** | **Minutes/day** | **Hours/day** | **Don’t know** |
| --- | --- | --- | --- |
| During the last 7 days, how much time did you spend sitting on a week day? |  |  | O |

**A2: Questions related to your sports activities**

The following questions will ask you about the time you spent in sport activities in a typical week. Please answer the following questions without considering light intensity activities frequently performed during daily life (e.g., self-care, washing dishes, using light tools at a desk) or activities of very short duration (e.g., taking out trash, walking to parking lot at store or office).

Think about all the moderate-intensity aerobic activities you do in a typical week. Moderate-intensity activities are activities that require moderate physical effort and cause small increases in breathing or heart rate such as brisk walking.

| **Question 8**** | **1** | **2** | **3** | **4** | | **5** | **6** | **7** |
| --- | --- | --- | --- | --- | --- | --- | --- | --- |
| In a typical week, on how many days do you do moderate-intensity aerobic related activities? | O | O | O | O | | O | O | O |
|  | O No moderate-intensity aerobic activities  Skip to question 10 | | | | | | | |
| **Question 9**** | **Minutes/day** | | | | **Hours/day** | | | |
| How much time do you spend doing such moderate-intensity aerobic related activities on such a typical day? |  | | | |  | | | |

Think about all the vigorous-intensity aerobic activities you do in a typical week. Vigorous-intensity activities are activities that causes rapid breathing and a substantial increase in heart rate such as jogging or running.

| **Question 10**** | **1** | **2** | **3** | **4** | | **5** | **6** | **7** |
| --- | --- | --- | --- | --- | --- | --- | --- | --- |
| In a typical week, on how many days do you do vigorous-intensity aerobic related activities? | O | O | O | O | | O | O | O |
|  | O No vigorous-intensity aerobic activities  Skip to question 12 | | | | | | | |
| **Question 11**** | **Minutes/day** | | | | **Hours/day** | | | |
| How much time do you spend doing such vigorous-intensity aerobic related activities on such a typical day? |  | | | |  | | | |

Think about all the vigorous-intensity aerobic activities you do in order to maintain or increase muscular strength. Muscle-strengthening activities include a progressive weight-training program, weight bearing calisthenics, stair climbing, and similar resistance exercises that use the major muscle groups.

| **Question 12**** | **1** | **2** | **3** | **4** | **5** | **6** | **7** |
| --- | --- | --- | --- | --- | --- | --- | --- |
| In a typical week, on how many days do you do strength-related activities or a strength training? | O | O | O | O | O | O | O |
|  | O No muscle-strengthening activities | | | | | | |

****** **Self-constructed item**

**B: Questions related to your exercise motivation**

We are interested in finding out your reasons for being be active in sport activities or not. Please answer each question according to the scale below. Indicate to what extent the following questions corresponds with your situation. There are no right or wrong answers and also no “trick questions”.

***Why are you (not) currently engaged in sport activities?***

|  | **I totally agree** | **I agree** | | | **I don’t no** | | **I disagree** | | **I totally disagree** |
| --- | --- | --- | --- | --- | --- | --- | --- | --- | --- |
|  | **1** | **2** | | | **3** | | **4** | | **5** |
| I don’t see the point in exercising | O | O | | | O | | O | | O |
| I do this activity but I am not sure if it is worth it | O | O | | | O | | O | | O |
| I don’t see why I should have to exercise | O | O | | | O | | O | | O |
| I think that exercising is a waste of time | O | O | | | O | | O | | O |
| I used to have good reasons for doing sports, but now I am asking myself if I should continue doing it | O | O | | | O | | O | | O |
| I don’t know anymore; I have the impression that I am incapable of succeeding in this sport | O | O | | | O | | O | | O |
| I can’t see why I should bother exercising | O | O | | | O | | O | | O |
| I often ask myself; I can’t seem to achieve the goals that I set for myself | O | O | | | O | | O | | O |
| There may be good reasons to do this activity, but personally I don’t see any | O | O | | | O | | O | | O |
| It is not clear to me anymore; I don’t really think my place is in sport | O | O | | | O | | O | | O |
| I don’t know; I don’t see what this activity brings me | O | | O | O | | O | | O | |
| I do this activity, but I am not sure it is a good thing to pursue it | O | | O | O | | O | | O | |

**C: Questions related to your attitude towards a physically active lifestyle**

We are interested in finding out your attitude towards a physically active lifestyle. Please answer each question according to the scale below. Indicate to what extent the following questions corresponds with your situation.

|  | **I totally agree** | **I agree** | **I don’t no** | **I disagree** | **I totally disagree** |
| --- | --- | --- | --- | --- | --- |
|  | **1** | **2** | **3** | **4** | **5** |
| Physical activity is part of my daily routine****** | O | O | O | O | O |
| I usually drive or ride short distances rather than walk | O | O | O | O | O |
| It is hard for me to find the time to exercise on a regular basis | O | O | O | O | O |
| When I exercise or play sports, I feel self-conscious about the way I look to others | O | O | O | O | O |
| It is hard for me to stick to a regular schedule of physical activity | O | O | O | O | O |
| I usually use the lift and/or the escalator rather than the stairway****** | O | O | O | O | O |
| I prefer leisure-time-activities, which are not connected with physical activity (for example computer games, movies, televisions-series etc.)****** | O | O | O | O | O |
| I think regular sport lessons at school are unnecessary****** | O | O | O | O | O |
| I need a lot of motivation and it costs me quite an effort to perform physical activities regularly****** | O | O | O | O | O |
| Physical activities respectively sport is an essential part of my life and belongs to a fulfilled life****** | O | O | O | O | O |

****** **Self-constructed item**

**D: Questions related to your exercise knowledge**

Please answer the following open-ended questions using keywords or short sentences. Please answer all five questions without any help or tools.

|  | **Free answers** | | | | |
| --- | --- | --- | --- | --- | --- |
| At least how many days per week a person at your age must perform moderate-intensity endurance physical activity to promote health?****** |  |  |  |  |  |
| At least how many minutes per day a person at your age must perform moderate-intensity physical activity to promote health?****** |  |  |  |  |  |
| At least how many days per week a person at your age must perform strength-based physical activity to maintain or increase muscular strength?****** |  |  |  |  |  |
| At least how many minutes must a single physical activity session last to promote health?****** |  |  |  |  |  |
| At least how many minutes must a single physical activity session last to promote health?****** |  |  |  |  |  |

****** **Self-constructed item**

**E: Questions related to your exercise self-confidence/self-efficacy**

We are interested in finding out your exercise self-confidence/self-efficacy. Please answer each question according to the scale below. Indicate to what extent the following questions corresponds with your situation.

|  | **I totally agree** | | **I agree** | **I don’t no** | | **I disagree** | | **I totally disagree** | |
| --- | --- | --- | --- | --- | --- | --- | --- | --- | --- |
|  | **1** | **2** | | | **3** | | **4** | | **5** |
| Overall, I see myself as robust and strong | O | O | | | O | | O | | O |
| There is something wrong with my physical appearance | O | O | | | O | | O | | O |
| I am healthy | O | O | | | O | | O | | O |
| Sometime I feel a boundless energy inside myself | O | O | | | O | | O | | O |
| My physical imperfections adversely affect my daily life | O | O | | | O | | O | | O |
| My body bothers me often | O | O | | | O | | O | | O |
| I feel myself full of power | O | O | | | O | | O | | O |
| I am dissatisfied with my physical appearance | O | O | | | O | | O | | O |
| Often I feel an excessive erotic tension inside myself | O | O | | | O | | O | | O |
| I would like another body | O | O | | | O | | O | | O |
| Sometimes I feel disgusted by my own body | O | O | | | O | | O | | O |
| I am full of powerful tension | O | O | | | O | | O | | O |
| I don’t like photos from myself | O | O | | | O | | O | | O |
| I feel myself physically fit | O | O | | | O | | O | | O |
| My physical deficiencies bother me a lot | O | O | | | O | | O | | O |
| I spend a lot of time thinking of my sexual desires | O | O | | | O | | O | | O |
| I am very confident to my physical talents | O | O | | | O | | O | | O |
| Sometime I have the desire to look differently | O | O | | | O | | O | | O |
| I feel like a stranger in my own body | O | O | | | O | | O | | O |
| I like to dance wildly | O | O | | | O | | O | | O |

***Certain barriers make it hard to begin exercising.***

***How sure are you that you can begin exercising regularly?***

**I am sure that...**

|  | **I totally agree** | | **I agree** | **I don’t no** | | **I disagree** | | **I totally disagree** | |
| --- | --- | --- | --- | --- | --- | --- | --- | --- | --- |
|  | **1** | | **2** | **3** | | **4** | | **5** | |
| I can change to a physically active life  style | O | O | | | O | | O | | O |
| I can be physically active once a week | O | O | | | O | | O | | O |
| I can be physically active at least 3 times  a week for 30 minutes | O | O | | | O | | O | | O |

**It is always hard to get started. How sure are you that you can start exercising regularly?**

***I am sure I can start being physically active immediately, even if...***

|  | **I totally agree** | **I agree** | **I don’t no** | **I disagree** | **I totally disagree** |
| --- | --- | --- | --- | --- | --- |
|  | **1** | **2** | **3** | **4** | **5** |
| ...I initially have to reconsider my views  on physical activity | O | O | O | O | O |
| ...the planning for this is very laborious | O | O | O | O | O |
| ...I have to force myself to start  immediately | O | O | O | O | O |
| ...I have to push myself | O | O | O | O | O |

**It is important to stay physically active. Are you confident you can manage that?**

***I am sure I can keep being physically active regularly, even if...***

|  | **I totally agree** | **I agree** | **I don’t no** | **I disagree** | **I totally disagree** |
| --- | --- | --- | --- | --- | --- |
|  | **1** | **2** | **3** | **4** | **5** |
| ... it takes me long to make it a habit | O | O | O | O | O |
| ...I am worried and troubled | O | O | O | O | O |
| ...I don’t see success at once | O | O | O | O | O |
| ...I am tired | O | O | O | O | O |
| ...I am stressed out | O | O | O | O | O |
| ...I feel tense | O | O | O | O | O |
| ...my blood pressure doesn’t improve  immediately | O | O | O | O | O |
| ...I won’t get social support for my first  attempts | O | O | O | O | O |
| ...I have to start all over again several  times until I succeed | O | O | O | O | O |
| ...my partner/ family isn’t physically  active | O | O | O | O | O |
| ...my cholesterol doesn’t improve  immediately | O | O | O | O | O |

**In spite of good intentions, smaller or larger relapses may occur. Imagine you stopped exercising for some time. How confident are you about restarting exercises?**

***I am sure I can be physically active again regularly, even if…***

|  | **I totally agree** | **I agree** | **I don’t no** | **I disagree** | **I totally disagree** |
| --- | --- | --- | --- | --- | --- |
|  | **1** | **2** | **3** | **4** | **5** |
| ...I postpone my plans several times | O | O | O | O | O |
| ...I am not able to pull myself together  sometimes | O | O | O | O | O |
| ...I have already paused for several  weeks | O | O | O | O | O |

**This is the end of the questionnaire, thank you for participating and supporting our research in the field of Physical Literacy.**
