# Supplementary material for: Holistic physical exercise training improves physical literacy among physically inactive adults: a pilot intervention study
Source: BMC Public Health. 2019 Apr 11;19:393. doi: 10.1186/s12889-019-6719-z (PMC6458734; doi:10.1186/s12889-019-6719-z)
Supplement: Supplementary file 2 — Running/walking - physical activity knowledge transfer (DOCX 157 kb) [file 12889_2019_6719_MOESM2_ESM.docx]

**Example of a running/walking game, designed to mediate cognitive elements of exercising and physical activity**

**Title: Physical Activity Puzzle-Run/Walk**

**Description:** Starting at point A, participants had to run or walk through an obstacle course (see Fig 1). Boxes containing puzzle pieces (one for each participant) were placed on a desk or something similar at point B. Each time participants ran or walked through the course and reached point B, they were allowed to collect exactly one puzzle piece. Subsequently, they had to carry the piece of the puzzle through the course back to point A for assembly. To ensure equality in terms of different physical capabilities of all participants, the boxes also contained some blank puzzle pieces, which did not contribute to the completion of the puzzle. The game ended after the first six participants had completed the puzzle. All applied puzzles were related to information on exercise and physical activity (e.g. national physical activity guidelines, see Fig 2). If necessary (e.g. due to a large heterogeneity of participants’ physical capabilities), two different obstacle course were used, an easy and a more difficult one.


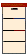

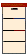

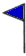

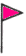


**A**

**B**


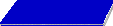

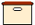

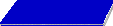

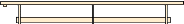

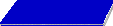

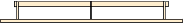





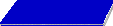

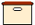

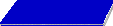

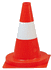

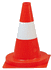

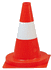

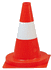

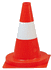

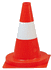

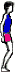

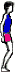

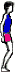

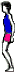

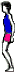

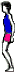


**Start/End**

**Boxes of puzzle pieces**

[
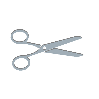
](https://www.google.at/url?sa=i&rct=j&q=&esrc=s&source=images&cd=&cad=rja&uact=8&ved=2ahUKEwiuzofCvbfdAhWHKlAKHZArDyIQjRx6BAgBEAU&url=https%3A%2F%2Fpixabay.com%2Fde%2Fscheren-symbol-vektor-clipart-2337264%2F&psig=AOvVaw0kkfPXHcHY30hUb4KaE12w&ust=1536910921768078)[
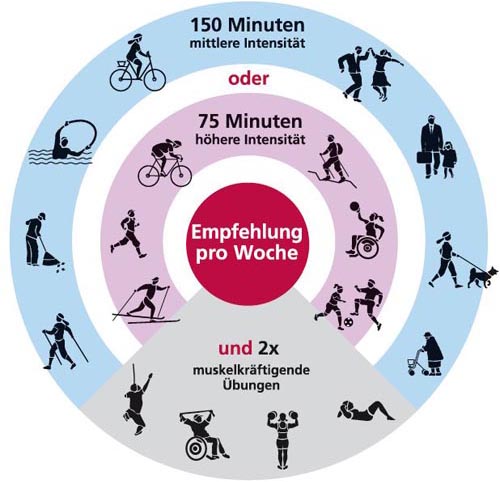
](https://www.google.at/url?sa=i&rct=j&q=&esrc=s&source=images&cd=&cad=rja&uact=8&ved=2ahUKEwjhyq22vLfdAhVOJVAKHRoLBAIQjRx6BAgBEAU&url=http%3A%2F%2Fwww.bewegungskompetenzen.at%2Fffoe%2Findex.php%2Fbewegungsempfehlungen&psig=AOvVaw0wpHEVn81QarASjw5PQj1T&ust=1536910633267869)

**Fig 1. Example of an obstacle course (illustrations: © Rolf Dober)**

**Fig 2. Example of an applied puzzle**

**Image source:** Titze S, Ring-Dimitriou S, Schober PH, Halbwachs C, Samitz G, Miko HC, et al. Österreichische Empfehlungen für gesundheitswirksame Bewegung. In: Gesundheit Österreich GmbH/Geschäftsbereich Fonds Gesundes Österreich, editors. 2012. <http://fgoe.org/sites/fgoe.org/files/2017-10/2012-10-17.pdf>. Accessed 10 Sep 2018.
