# Supplementary material for: Holistic physical exercise training improves physical literacy among physically inactive adults: a pilot intervention study
Source: BMC Public Health. 2019 Apr 11;19:393. doi: 10.1186/s12889-019-6719-z (PMC6458734; doi:10.1186/s12889-019-6719-z)
Supplement: Supplementary file 3 — Descriptive characteristics of the IG subsamples (DOCX 15 kb) [file 12889_2019_6719_MOESM3_ESM.docx]

**Participant characteristics of IG subsamples and control group**

| **Variables** | **IG-GP**  (n = 16) | **IG-nGP**  (n = 15) | **CG**  (n = 30) |
| --- | --- | --- | --- |
| **Gender (n females)** | 12 (75%) | 13 (87%) | 24 (80%) |
| **Age (years)** | 53 ± 9 | 33 ± 15**^**^** | 45 ± 11 |
| **Anthropometry** |  |  |  |
| Height (cm) | 169 ± 9 | 166 ± 6 | 169 ± 7 |
| Weight (kg) | 86 ± 25^*^ | 63 ± 9 | 72 ± 15 |
| BMI (kg/m²) | 30 ± 9^*^ | 23 ± 3 | 25 ± 4 |
| **Education level** |  |  |  |
| Compulsory school (n) | 5 (31%) | 0 (0%) | 3 (10%) |
| Apprenticeship/  Professional school degree (n) | 7 (44%) | 5 (33%) | 15 (50%) |
| School leaving examination  (A-Level) (n) | 3 (19%) | 9 (60%) | 8 (27%) |
| Grad/professional degree (n) | 1 (6%) | 1 (7%) | 4 (13%) |
| **Chronic diseases (n yes)** | 10 (63%)^*^ | 2 (13%) | 6 (20%) |
| **Physical Literacy (%)** | 55 ± 10 | 64 ± 9^***^ | 59 ± 10 |
| Physical activity behaviour (%)^a^ | 25 (25) | 25 (25) | 25 (25) |
| Attitude (%) | 59 ± 18 | 67 ± 18 | 62 ± 17 |
| Motivation (%) | 74 ± 14 | 82 ± 19 | 74 ± 14 |
| Knowledge (%) | 54 ± 20 | 56 ± 11 | 57 ± 22 |
| Self-confidence/Self-efficacy (%) | 62 ± 13 | 74 ± 9^***^ | 66 ± 11 |

Values are given as mean ± SD or as frequency (%); *IG-GP*: IG participants, recruited from GP. *IG-nGP*: IG participants not recruited from GP. *CG*: Control group. *BMI*: body mass index; **^*^**different from IG-nGP and CG, p ≤ 0.01; ^**^different from IG-GP and CG, p ≤ 0.01; ^***^different from IG-GP, p ≤ 0.05;

^a^Given as median ± IQR, because ordinal scaled
